# Localization, regulation and function of Type II phosphatidylinositol 5-phosphate 4-kinases

**DOI:** 10.1016/j.advenzreg.2009.10.006

**Published:** 2010

**Authors:** Jonathan H. Clarke, Michael Wang, Robin F. Irvine

**Affiliations:** Department of Pharmacology, University of Cambridge, Tennis Court Road, Cambridge CB2 1PD, UK

## Introduction

**Fig. 1:**
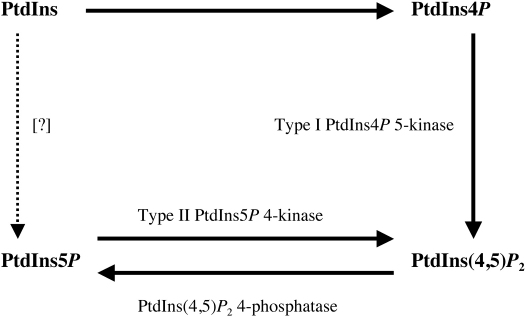
The PtdIns*P* kinases. The inositol lipids and kinases that are the principal topics of discussion in this review are illustrated here. EC Numbers: Type I PtdIns4*P* 5-kinase, EC 2.7.1.68; Type II PtdIns5*P* 4-kinase, EC 2.7.1.149; PtdIns(4,5)*P*_2_ 4-phosphatase, EC 3.1.3.78.

The variety of form and function in inositides (inositol lipids and phosphates) stems largely from the degree and isomeric specificity of phosphorylation of their common inositol ring. Thus their synthesis, and its differential regulation, lie primarily in the large family of kinases that phosphorylate the inositol ring. In a previous review in this publication (Irvine et al., 2006) we addressed the inositol phosphate kinases, with a specific focus on the Ins(1,4,5)*P*_3_ 3-kinase family. Here we turn to the inositol lipid kinases, with a specific focus on the phosphatidylinositol phosphate kinases. These are the family of enzymes that synthesize phosphatidylinositol 4,5-bisphosphate (PtdIns(4,5)*P*_2_) (Fig. 1). The Type I enzymes (EC 2.7.1.68) are now known to catalyze the major route of PtdIns(4,5)*P*_2_ synthesis, the 5-phosphorylation of PtdIns4*P* (Rameh et al., 1997), and are here abbreviated as Type I PtdIns4*P* 5-kinases (PtdIns4*P* 5-kinases Iα, Iβ and Iγ). The Type II enzymes (EC 2.7.1.149) are 4-kinases (Rameh et al., 1997), whose preferred substrate (Rameh et al., 1997; Morris et al., 2000; Roberts et al., 2005) is PtdIns5*P*. They will be abbreviated here as PtdIns5*P* 4-kinases IIα, IIβ and IIγ.

## Type II PtdIns5*P* 4-kinases

### Type II PtdIns5*P* 4-kinases – basic functions

Although there has been some discussion about the potential functions of the Type II enzymes, there is an emerging consensus that their major function is to regulate the levels of their substrate, PtdIns5*P*. The amount of PtdIns(4,5)*P*_2_ that they will synthesize relative to the Type I enzymes is likely to be small (mostly because PtdIns5*P* is present at much lower levels than PtdIns4*P* (Roberts et al., 2005)). Also, the recent discovery and cloning in Majerus' lab of two isoforms of PtdIns(4,5)*P*_2_ 4-phosphatase (EC 3.1.3.78) (Ungewickell et al., 2005; Zou et al., 2007), one of which is partly nuclear (Zou et al., 2007) (see below for the significance of this) generates a very plausible cycle for the generation of PtdIns5*P* (by a PtdIns(4,5)*P*_2_ 4-phosphatase) and its removal (by a Type II PtdIns5*P* 4-kinase). Lecompte et al. (2008) have suggested an interesting evolutionary pathway for the appearance of this cycle – that PtdIns5*P* generation and removal may occur by different routes involving different combinations of enzymes (e.g. from PtdIns *via* Type III PtdIns3*P* 5-kinase and myotubularin) and that the PtdIns(4,5)*P*_2_ 4-phosphatase/Type II PtdIns5*P* 4-kinase ‘cycle’ may be the latest of these to evolve, and is confined to metazoans. It is worth noting in this context that the significance of a PTEN homologue that is a 5-phosphatse (EC 3.1.3.36), but very specific for PtdIns5*P* (Pagliarini et al., 2004), remains to be explored fully – is this an alternative way of removing PtdIns5*P*, and if so, how does it fit in with the Type II PtdIns5*P* 4-kinases?

### PtdIns5*P*

The functions of PtdIns5*P* are being actively explored, and it seems likely that our list of these is far from complete. This is a pertinent place to point out an error in our description of the mass assay for PtdIns5*P*, which we described in Roberts et al. (2005). This was a refinement of the original assay (Morris et al., 2000), and included, after loading the neomycin beads with lipids, a prior rinse with ‘50 mM Ammonium formate’, before two elutions of inositol lipids with ‘2 M TEAB’ (Triethylammonium bicarbonate). Both these two solutions were described as if they were made up in water, but they should of course have been described as being mixed with chloroform and methanol as with the other bead-elution solutions used in this protocol. Thus they are respectively: CHCl_3_:CH_3_OH:Ammonium Formate 5:10:2 by volume (final concentration of formate 50 mM); and CHCl_3_:CH3OH:2 M TEAB 5:10:2 by volume (final concentration of TEAB 0.55 M). This latter solution (with slightly altered CHCl_3_:CH_3_OH:2 M TEAB proportions), is correctly described by Zou et al. (2007), who also successfully used a more widely available type of glass bead for this experimental procedure.

To return to PtdIns5*P* functions, in the cytosol its principal effect has been suggested to be to increase Akt activation (Pendaries et al., 2006), perhaps by inhibiting its dephosphorylation (Ramel et al., 2009) or the dephosphorylation of PtdIns(3,4,5)*P*_3_ (Carricaburu et al., 2003). Recently two putative PtdIns5*P* effectors, Dok-1 and Dok-2 were described in T cells (Guittard et al., 2009), and the significance of these will be an interesting area for future exploration. A better understood aspect of PtdIns5*P* function is in the nucleus, where ING-2 has been identified as an effector (Gozani et al., 2003). Divecha's group have put together a convincing case that PtdIns5*P* 4-kinase IIβ regulates the levels of nuclear PtdIns5*P* (Jones et al., 2006), whereby stressing cells activates p38 MAP kinase, which phosphorylates PtdIns5*P* 4-kinase IIβ on two Ser residues causing its inhibition, and thus PtdIns5*P* increases. This complements the demonstration that stress increases the nuclear localization of the PtdIns5*P*-generating enzyme Type I PtdIns(4,5)*P*_2_ 4-phosphatase (Zou et al., 2007). So, what regulates the nuclear localization of PtdIns5*P* 4-kinase IIβ, and is this the only Type II enzyme involved? We have made some interesting observations in this context recently, which we will briefly summarize here.

### PtdIns5*P* 4-kinases IIα and IIβ

Although there have been some indications that endogenous PtdIns5*P* 4-kinase IIα may be partly nuclear (Divecha et al., 1993; Boronenkov et al., 1998), extensive studies on transfected cells have shown that the IIα and IIβ isoforms are respectively cytosolic and nuclear, with the latter being localized by a unique nuclear localization sequence consisting of an acidic α-helix (Ciruela et al., 2000; Clarke et al., 2007). Moreover, tagging the endogenous PtdIns5*P* 4-kinase IIβ by genomic tagging in DT40 cells (see below) confirmed that the endogenous enzyme appeared to be entirely nuclear, within the limits of the cell fractionation procedure employed (Richardson et al., 2007). However, a nuclear localization for PtdIns5*P* 4-kinase IIβ sat at odds with the phenotype of the knockout mouse (Lamia et al., 2004) complemented by transfection studies with PtdIns5*P* 4-kinase IIβ, both of which pointed to a predominantly cytoplasmic role for this enzyme (Carricaburu et al., 2003).

We have recently used genomic tagging in DT40 cells to begin to resolve some of these contradictions and to throw a new light on the relationship between these two Type II PtdIns5*P* 4-kinase isoforms (M.W., N. Bond, J. Richardson, K. Lilley, R.F.I. and J.H.C unpublished observations). In brief, we have found that PtdIns5*P* 4-kinases IIα and IIβ heterodimerize, such that a significant proportion of IIα is nuclear. Moreover, the enzymic activity of the IIα is much greater than that of IIβ, so it is possible that a, or the, major function of the IIβ isoform is simply to target the IIα to the nucleus. This is particularly interesting in the light of the evidence, based on mRNA levels, that in most tissues, indeed, in all that were studied except spleen, the IIβ isoform is more highly expressed than the IIα (Clarke et al., 2008).

Note that spleen is a site of synthesis of B cells (of which DT40s are, indirectly, an example), and T cells, so it is interesting that, as described above, Guittard et al. (2009) have recently discovered two new, cytoplasmic, putative PtdIns5*P* effectors in T cells. The spleen is also the site of synthesis of erythrocytes and platelets, from which PtdIns5*P* 4-kinase IIα was first purified and cloned by Boronenkov and Anderson (1995) and Divecha et al. (1995) respectively. Another intriguing link between erythrocytes and PtdIns5*P* 4-kinase IIα has emerged recently in a pair of α-thalassemic twins with very different β-globin gene expression levels, in which the only other gene showing a similar disparity in expression in their reticulocytes was PtdIns5*P* 4-kinase IIα (Wenning et al., 2009).

This emphasis on PtdIns5*P* 4-kinase IIα in blood cells makes a contrast with all other tissues so far investigated in this context, where PtdIns5*P* 4-kinase IIβ mRNA is dominant over that for PtdIns5*P* 4-kinase IIα (Clarke et al., 2008). It throws open the possibility that in many tissues most PtdIns5*P* 4-kinase IIα is nuclear, and this will be especially so in muscle and liver (Clarke et al., 2008). This in turn sheds an alternative light on the experiments discussed above where PtdIns5*P* 4-kinase IIβ levels were manipulated (Carricaburu et al., 2003; Lamia et al., 2004), in that perhaps the major effect of decreasing PtdIns5*P* 4-kinase IIβ is to increase cytosolic IIα because there is no IIβ to take it to the nucleus. Of course we don't yet know if IIα/IIβ heterodimers are localized within the nucleus in the same place as IIβ/IIβ homodimers, nor whether they have distinct functions, and overall our discovery of this heterodimerization asks more questions than it answers. But that is what makes it interesting.

### PtdIns5P 4-kinase IIγ

This has been the ‘Cinderella’ member of the family until recently, and we have been investigating some of its basic biology and biochemistry, which we summarize here.

**Fig. 2:**
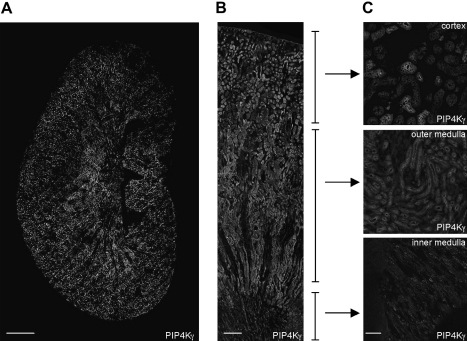
PtdIns5*P* 4-kinase IIγ expression in the adult mouse kidney. An antibody specific to PtdIns5*P* 4-kinase IIγ was used in tissue immunohistochemistry to identify endogenous expression in kidney sections; for details see (Clarke et al., 2008). A) PtdIns5*P* 4-kinase IIγ in a whole saggital kidney section (scale bar = 1 mm). B) PtdIns5*P* 4-kinase IIγ in a representative strip showing differential PtdIns5*P* 4-kinase IIγ expression from outer capsule to calyx (scale bar = 250 μm). C) Inset slides show PtdIns5*P* 4-kinase IIγ in the cortex, outer and inner medulla. The PtdIns5*P* 4-kinase IIγ signal is restricted to specific cells within these regions (scale bar = 40 μm).

When first cloned by Itoh et al. (1998), PtdIns5*P* 4-kinase IIγ was described as being highly expressed in kidney, localized to the endoplasmic reticulum, and phosphorylated when cells were stimulated by mitogens. We have confirmed that kidney expresses PtdIns5*P* 4-kinase IIγ more highly than any other tissue – as judged by mRNA levels, kidney is unique in expressing more PtdIns5*P* 4-kinase IIγ than PtdIns5*P* 4-kinase IIα and IIβ combined (Clarke et al., 2008). Even more striking is the localization of expression within the kidney, as PtdIns5*P* 4-kinase IIγ is largely confined to epithelial cells in the thick ascending limb and the intercalated cells of the collecting duct (Fig. 2 and Clarke et al., 2008).

The distribution of PtdIns5*P* 4-kinase IIγ in nervous tissue, the other type of tissue in which it is highly expressed (Clarke et al., 2008), shows a similar restricted and well defined expression in the brain and also the spinal cord (Clarke et al., 2009). This expression is limited to neurons, particularly the cerebellar Purkinje cells, pyramidal cells of the hippocampus, large neuronal cell-types in the cerebral cortex including pyramidal cells, and mitral cells in the olfactory bulb, but is not expressed in cerebellar, hippocampal formation or olfactory bulb granule cells (Clarke et al., 2009).

These distinct and specific expression patterns in brain and kidney will eventually tell us something of its function, though at present this is difficult to guess. However, two other properties of PtdIns5*P* 4-kinase IIγ point a way forward in understanding what it does. Firstly, in both kidney (Clarke et al., 2008) and brain (Clarke et al., 2009) it shows the same intracellular distribution: it appears to be associated with vesicles, which in kidney epithelial cells are concentrated towards the secreting end of the cells (Clarke et al., 2008).

We do not yet know the nature of these vesicles. In neurons PtdIns5*P* 4-kinase IIγ shows a partial colocalization with markers of cellular compartments of the endomembrane trafficking pathway (Clarke et al., 2009), and transfection experiments with mildly permeabilized HeLa cells showed a partial colocalization with Golgi markers such as GM130 and golgin 160, as well as the endosomal marker EEA1, but after more extensive cell permeabilization these correlations were decreased (Clarke et al., 2009). In transfected kidney cell lines (Clarke et al., 2008), PtdIns5*P* 4-kinase IIγ was again partially colocalized with GM130, but not with endoplasmic reticulum or ERGIC markers, and the GM130 relationship survived Brefeldin A treatment of the cells.

Together these data suggest a close association of PtdIns5*P* 4-kinase IIγ with vesicular cell trafficking linked with the Golgi apparatus. In the kidney this would be associated with the business of inserting and removing plasma membrane transporters and channels, and in the brain either trafficking of vesicles along neuronal processes or perhaps the delivery/insertion of channels and transporters to their correct locations. PtdIns(4,5)*P*_2_ is of course known to be associated with the regulation of activity and trafficking of ion channels and transporters, but returning to the low levels of PtdIns5*P* compared to PtdIns4*P* in cells plus the evidence discussed above for possible roles of PtdIns5*P* in other cellular functions, we think it much more likely that PtdIns5*P* is the active molecule in whatever vesicular processing/transporting events PtdIns5*P* 4-kinase IIγ is regulating.

Secondly, there is another aspect of PtdIns5*P* 4-kinase IIγ biochemistry which may point to how it functions – actually it is two properties that suggest the same thing. PtdIns5*P* 4-kinase IIγ is, when bacterially expressed, catalytically close to inactive (Clarke et al., 2008); it has even less activity than PtdIns5*P* 4-kinase IIβ (above). But when immunoprecipitated from eukaryotic cells, PtdIns5*P* 4-kinase IIγ does show some catalytic activity, which can be accounted for by its association with PtdIns5*P* 4-kinase IIα, an association that we have demonstrated directly *in vitro* (Clarke et al., 2008). Thus we can see a parallel with the PtdIns5*P* 4-kinase IIα/IIβ heterodimerization that we have discussed above, and our current working hypothesis for PtdIns5*P* 4-kinase IIγ is that it is associated with a sub-population of vesicles in the cell trafficking system, and by heterodimerization with PtdIns5*P* 4-kinase IIα it targets the latter enzyme to the relevant vesicles, where it regulates PtdIns5*P* levels for a function yet to be defined (see above). We should note also that there is still the possibility that by associating with a Type I PtdIns4*P* 5-kinase activity (Hinchliffe et al., 2002), PtdIns5*P* 4-kinase IIα may also target one of these enzymes to the relevant vesicles (discussed further below), so PtdIns(4,5)*P*_2_ might yet be a relevant signal in this context too.

So our recent data are painting a different picture of Type II PtdIns5*P* 4-kinase function and regulation from that which we have had before. We can suggest the possibility that PtdIns5*P* 4-kinase IIα is the (possibly the only significantly) active enzyme of the three isoforms, and that it may have its own individual function(s) in cells. Additionally it can be targeted by the other isoforms, to the nucleus by PtdIns5*P* 4-kinase IIβ, or to trafficking/secretory vesicles by PtdIns5*P* 4-kinase IIγ. This picture paints a remarkable (and we suspect unique) relationship between three isoforms of the same lipid kinase family.

### Association between Type I and Type II PtdIns*P* kinases

Before leaving the subject of the heterodimerization of Type II PtdIns5*P* 4-kinases, another dimerization (or at least, association) needs discussing in this context, which is the association of PtdIns5P 4-kinase IIα with Type I PtdIns4*P* 5-kinases. This binding is clearly documented both with transfected and endogenous Type I enzymes (Hinchliffe et al., 2002). We do not yet know which structural domains of either partner (Type I or II) are involved in this interaction. The regions involved in heterodimerization between PtdIns5*P* 4-kinases IIα, IIβ, and IIγ is fairly certain: the β isoform is a homodimer in the crystals used for structural analysis, with the interaction between the monomers being due to two opposing β-pleated sheets (Rao et al., 1998), and this part of the enzyme is identical in all three isoforms. But a crucial question is whether a hetero (or homo) dimerized Type II PtdIns5*P* 4-kinase is capable of also associating with a Type I PtdInd4*P* 5-kinase, or, put another way, does the Type I/II association involve the same region of the enzymes so that a Type II enzyme is compelled to associate either with a Type I or a Type II PtdIns*P* kinase, but not with both?

This is not a trivial question, as one of the interesting ideas that we discussed above is that the localization of the highly active Type IIα PtdIns5*P* 4-kinase may be governed entirely by the relative levels of expression of the IIβ and IIγ isoforms, levels that can vary greatly between tissues (Clarke et al., 2008). If we have to factor into that idea the relative levels of the three Type I PtdIns4*P* 5-kinases, each of which can interact with the Type IIα PtdIns5*P* 4-kinase (Hinchliffe et al., 2002) (and we do not yet know if Type IIβ or IIγ can interact with Type I activities), we have a very complex picture. Indeed, such a proposal would seem to make a nonsense of the above idea that relative levels of the three Type II isoforms has physiological relevance in dictating their localization, and the whole process of PtdIns*P* kinases associating would have to be tightly and complicatedly regulated. We should add that, when pulling down PtdIns5*P* 4-kinase IIβ from DT40 cells (above) we did not detect any Type I isoforms by mass spectroscopy, so any Type I/II interaction must be of lower affinity than Type II/II interactions. Overall we feel that a more likely idea is that the domains by which Type II enzymes interact with each other (Rao et al., 1998) are different from those that PtdIns5*P* 4-kinase IIα uses to associate with Type I enzymes, and so a Type II PtdIns*P* 4-kinase homo or heterodimer can additionally associate with a (two?) Type I PtdIns4*P* 5-kinase enzyme molecule(s). The functional consequences of this latter association still elude us.

## Summary

Our recent studies of the Type II PtdIns5*P* 4-kinases have revealed that the Type IIα isoform is very much more active than the IIβ or IIγ isoforms, and that it can (and does physiologically) heterodimerize with them. This suggests the idea that the Type IIα enzyme is targeted to the nucleus (by dimerization with Type IIβ), to secretory/transport vesicles (by dimerization with Type IIγ), or to the cytoplasm (as a homodimer), with the relative proportions of PtdIns5*P* 4-kinase activity at these localizations being regulated by the relative amounts of the three Type II isoforms expressed in any cell. The targeting to vesicles by PtdIns5*P* 4-kinase IIγ is likely to be of particular significance in epithelial cells in specific regions of the kidney tubules and in a sub-population of neurons in the brain and the spinal cord. The relationship between this dimerization between Type II PtdIns5*P* 4-kinase isoforms and the known ability of Type IIα PtdIns5*P* 4-kinase to associate with Type I PtdIns4*P* 5-kinases remains to be explored.

## References

[bib1] Boronenkov I.V., Anderson R.A. (1995). The sequence of phosphatidylinositol-4-phosphate 5-kinase defines a novel family of lipid kinases. J Biol Chem.

[bib2] Boronenkov I.V., Loijens J.C., Umeda M., Anderson R.A. (1998). Phosphoinositide signaling pathways in nuclei are associated with nuclear speckles containing pre-mRNA processing factors. Mol Biol Cell.

[bib3] Carricaburu V., Lamia K.A., Lo E., Favereaux L., Payrastre B., Cantley L.C. (2003). The phosphatidylinositol (PI)-5-phosphate 4-kinase type II enzyme controls insulin signaling by regulating PI-3,4,5-trisphosphate degradation. Proc Natl Acad Sci U S A.

[bib4] Ciruela A., Hinchliffe K.A., Divecha N., Irvine R.F. (2000). Nuclear targeting of the β isoform of Type II phosphatidylinositol phosphate kinase (phosphatidylinositol 5-phosphate 4-kinase) by its α-helix 7. Biochem J.

[bib5] Clarke J.H., Emson P.C., Irvine R.F. (2008). Localization of phosphatidylinositol phosphate kinase IIgamma in kidney to a membrane trafficking compartment within specialized cells of the nephron. Am J Physiol Renal Physiol.

[bib6] Clarke J.H., Emson P.C., Irvine R.F. (2009). Distribution and neuronal expression of phosphatidylinositol phosphate kinase IIγ in the mouse brain. J Comp Neurol.

[bib7] Clarke J.H., Richardson J.P., Hinchliffe K.A., Irvine R.F. (2007). Type II PIP kinases: location, regulation and function. Biochem Soc Symp.

[bib8] Divecha N., Rhee S.G., Letcher A.J., Irvine R.F. (1993). Phosphoinositide signalling enzymes in rat liver nuclei: phosphoinositidase C isoform beta 1 is specifically, but not predominantly, located in the nucleus. Biochem J.

[bib9] Divecha N., Truong O., Hsuan J.J., Hinchliffe K.A., Irvine R.F. (1995). The cloning and sequence of the C isoform of PtdIns4P 5-kinase. Biochem J.

[bib10] Gozani O., Karuman P., Jones D.R., Ivanov D., Cha J., Lugovskoy A.A. (2003). The PHD finger of the chromatin-associated protein ING2 functions as a nuclear phosphoinositide receptor. Cell.

[bib11] Guittard G., Gerard A., Dupuis-Coronas S., Tronchere H., Mortier E., Favre C. (2009). Cutting edge: Dok-1 and Dok-2 adaptor molecules are regulated by phosphatidylinositol 5-phosphate production in T cells. J Immunol.

[bib12] Hinchliffe K.A., Giudici M.L., Letcher A.J., Irvine R.F. (2002). Type IIalpha phosphatidylinositol phosphate kinase associates with the plasma membrane via interaction with type I isoforms. Biochem J.

[bib13] Irvine R.F., Lloyd-Burton S.M., Yu C.H.Y., Letcher A.J., Schell M.J. (2006). The regulation and function of inositol 1,4,5-trisphosphate 3-kinases. Adv Enzyme Regul.

[bib14] Itoh T., Ijuin T., Takenawa T. (1998). A novel phosphatidylinositol-5-phosphate 4-kinase (phosphatidylinositol- phosphate kinase IIgamma) is phosphorylated in the endoplasmic reticulum in response to mitogenic signals. J Biol Chem.

[bib15] Jones D.R., Bultsma Y., Keune W.J., Halstead J.R., Elouarrat D., Mohammed S. (2006). Nuclear PtdIns5P as a transducer of stress signaling: an in vivo role for PIP4Kbeta. Mol Cell.

[bib16] Lamia K.A., Peroni O.D., Kim Y.B., Rameh L.E., Kahn B.B., Cantley L.C. (2004). Increased insulin sensitivity and reduced adiposity in phosphatidylinositol 5-phosphate 4-kinase beta-/- mice. Mol Cell Biol.

[bib17] Lecompte O., Poch O., Laporte J. (2008). PtdIns5P regulation through evolution: roles in membrane trafficking?. Trends Biochem Sci.

[bib18] Morris J.B., Hinchliffe K.A., Ciruela A., Letcher A.J., Irvine R.F. (2000). Thrombin stimulation of platelets causes an increase in phosphatidylinositol 5-phosphate revealed by mass assay. FEBS Lett.

[bib19] Pagliarini D.J., Worby C.A., Dixon J.E. (2004). A PTEN-like phosphatase with a novel substrate specificity. J Biol Chem.

[bib20] Pendaries C., Tronchere H., Arbibe L., Mounier J., Gozani O., Cantley L. (2006). PtdIns(5)P activates the host cell PI3-kinase/Akt pathway during *Shigella flexneri* infection. EMBO J.

[bib21] Rameh L.E., Tolias K.F., Duckworth B.C., Cantley L.C. (1997). A new pathway for synthesis of phosphatidylinositol-4,5-bisphosphate. Nature.

[bib22] Ramel D., Lagarrigue F., Dupuis-Coronas S., Chicanne G., Leslie N., Gaits-Iacovoni F. (2009). PtdIns5P protects Akt from dephosphorylation through PP2A inhibition. Biochem Biophys Res Commun.

[bib23] Rao V.D., Misra S., Boronenkov I.V., Anderson R.A., Hurley J.H. (1998). Structure of type IIbeta phosphatidylinositol phosphate kinase: a protein kinase fold flattened for interfacial phosphorylation. Cell.

[bib24] Richardson J.P., Wang M., Clarke J.H., Patel K.J., Irvine R.F. (2007). Genomic tagging of endogenous type IIbeta phosphatidylinositol 5-phosphate 4-kinase in DT40 cells reveals a nuclear localisation. Cell Signal.

[bib25] Roberts H.F., Clarke J.H., Letcher A.J., Irvine R.F. (2005). Effect of lipid kinase expression and cellular stimuli on phosphatidylinositol 5-phosphate levels in mammalian cell lines. FEBS Lett.

[bib26] Ungewickell A., Hugge C., Kisseleva M., Chang S.C., Zou J., Feng Y. (2005). The identification and characterization of two phosphatidylinositol-4,5-bisphosphate 4-phosphatases. Proc Natl Acad Sci U S A.

[bib27] Wenning M.R., Mello M.P., Andrade T.G., Lanaro C., Albuquerque D.M., Saad S.O. (2009). PIP4KIIA and beta-globin: transcripts differentially expressed in reticulocytes and associated with High levels of Hb H in two Siblings with Hb H Disease. Eur J Haematol.

[bib28] Zou J., Marjanovic J., Kisseleva M.V., Wilson M., Majerus P.W. (2007). Type I phosphatidylinositol-4,5-bisphosphate 4-phosphatase regulates stress-induced apoptosis. Proc Natl Acad Sci U S A.

